# Whole genome sequencing of *Salmonella* Typhimurium illuminates distinct outbreaks caused by an endemic multi-locus variable number tandem repeat analysis type in Australia, 2014

**DOI:** 10.1186/s12866-016-0831-3

**Published:** 2016-09-15

**Authors:** Anastasia Phillips, Cristina Sotomayor, Qinning Wang, Nadine Holmes, Catriona Furlong, Kate Ward, Peter Howard, Sophie Octavia, Ruiting Lan, Vitali Sintchenko

**Affiliations:** 1 Centre for Infectious Diseases and Microbiology-Public Health, Westmead Hospital, Sydney, NSW Australia; 2 Marie Bashir Institute for Emerging Infectious Diseases and Biosecurity and Sydney Medical School, The University of Sydney, Sydney, NSW Australia; 3 NSW Enteric Reference Laboratory, Centre for Infectious Diseases and Microbiology Laboratory Services, Pathology West, Sydney, NSW Australia; 4 OzFood Net, Communicable Disease Branch, Health Protection, NSW Ministry of Health, Sydney, NSW Australia; 5 School of Biotechnology and Biomolecular Sciences, University of New South Wales, Sydney, NSW Australia

**Keywords:** *Salmonella* Typhimurium, Molecular epidemiology, Foodborne outbreaks, Whole genome sequencing, Public health surveillance

## Abstract

**Background:**

*Salmonella* Typhimurium (STM) is an important cause of foodborne outbreaks worldwide. Subtyping of STM remains critical to outbreak investigation, yet current techniques (e.g. multilocus variable number tandem repeat analysis, MLVA) may provide insufficient discrimination. Whole genome sequencing (WGS) offers potentially greater discriminatory power to support infectious disease surveillance.

**Methods:**

We performed WGS on 62 STM isolates of a single, endemic MLVA type associated with two epidemiologically independent, food-borne outbreaks along with sporadic cases in New South Wales, Australia, during 2014. Genomes of case and environmental isolates were sequenced using HiSeq (Illumina) and the genetic distance between them was assessed by single nucleotide polymorphism (SNP) analysis. SNP analysis was compared to the epidemiological context.

**Results:**

The WGS analysis supported epidemiological evidence and genomes of within-outbreak isolates were nearly identical. Sporadic cases differed from outbreak cases by a small number of SNPs, although their close relationship to outbreak cases may represent an unidentified common food source that may warrant further public health follow up. Previously unrecognised mini-clusters were detected.

**Conclusions:**

WGS of STM can discriminate foodborne community outbreaks within a single endemic MLVA clone. Our findings support the translation of WGS into public health laboratory surveillance of salmonellosis.

**Electronic supplementary material:**

The online version of this article (doi:10.1186/s12866-016-0831-3) contains supplementary material, which is available to authorized users.

## Background

*Salmonella* gastroenteritis is responsible for considerable disease burden in both developed and developing countries, with an estimated 93.8 million cases and 155,000 deaths each year [1]. The majority of these cases are foodborne [1–3], although transmission may occur directly from infected persons [4]. *Salmonella* is frequently identified as the aetiological agent in foodborne outbreaks in Australia, with *Salmonella enterica* subsp. *enterica* serovar Typhimurium (*S.* Typhimurium/STM) being the predominant serovar [5].

Timely subtyping of *Salmonella* and integration of laboratory findings into public health actions are critical in reducing delays in outbreak investigation [6]. In New South Wales (NSW), Australia, where STM accounts for around half of all culture-confirmed cases of salmonellosis [7], multi-locus variable number tandem repeat analysis (MLVA) has been used since 2006 to prospectively subtype STM and identify potential clusters. MLVA measures the variable length of five STM loci and has been considered faster and more discriminatory than other historical typing methods [7, 8]. However, common or endemic MLVA types may cause multiple outbreaks along with sporadic cases and a few selected MLVA types may represent a large portion of isolates observed in a geographical location [9].

Whole genome sequencing (WGS) has offered the ultimate discriminatory power with the potential to enhance epidemiological investigations and elucidate transmission pathways [9–11]. As WGS continually becomes quicker and more cost effective, and data quality improves [12–15] its potential to be used in routine epidemiological typing increases [15]. WGS has been recently used to understand outbreak sources and transmission patterns of other diseases, including enteric diseases (such as *Shigella sonnei* and enterohaemorrhagic *Escherichia coli*) [12, 14, 16–18]. Furthermore, WGS has the potential to discriminate between sporadic and outbreak isolates which may be indistinguishable by current methods of subtyping [14, 17, 19, 20].

Recently, the potential of WGS for characterising *Salmonella* outbreaks and differentiating outbreak and sporadic strains has been explored [15, 21] and the importance of correlating WGS and epidemiological data has been emphasised [15]. With increased understanding of the significance of genomic variability, there is potential to progress to near real-time, discriminatory genotyping of outbreak cases [21].

The primary aim of this study was to examine the utility of WGS in discriminating sporadic and outbreak-linked STM infections within the same endemic MLVA type. This has the potential to improve the accuracy of cluster definitions and timeliness of outbreak response. We report the WGS analysis of human and food STM isolates from two epidemiologically independent outbreaks of a single MLVA type in NSW during 2014, along with interspersed sporadic cases.

## Methods

### Isolate selection

Salmonellosis is a notifiable disease in Australia. Details of cases are maintained in the NSW jurisdictional Notifiable Conditions Information Management System (NCIMS) Database by the Communicable Disease Branch, Health Protection NSW. All case isolates presumptively identified as *Salmonella* by pathology service providers in NSW are forwarded to the NSW State Enteric Reference Laboratory at the Centre for Infectious Diseases and Microbiology, Institute for Clinical Pathology and Medical Research-Pathology West (ICPMR) in Sydney. Isolates undergo confirmatory testing, serotyping and MLVA-5 typing at ICPMR. Relevant food isolates from outbreak investigations are also typed and stored.

A common MLVA type (2-15-9-10-0212 or 3-17-10-11-523 according to the European CDC [22] and Australian conventions [23], respectively) that was known to have caused two food-borne outbreaks along with sporadic cases between January and May 2014 in metropolitan Sydney was selected for this study. STM of this MLVA type was responsible for 3.7 % of all STM infections in 2014. These isolates belonged to phage type DT135. The two outbreaks were dispersed in time and space such that sporadic cases occurred within two months of either or both outbreaks.

Each case isolate used in this study was classified as “outbreak”, “sporadic” or “secondary” by an epidemiologist at the Communicable Disease Branch based on information that was available for each case in NCIMS. This information was a case interview, a completed online questionnaire, and/or demographic information. Outbreak cases were those with documented exposure at one of the established outbreak venues. Sporadic cases were those with no documented exposure to one of these venues in the time frame that fitted with the incubation period for *Salmonella*. Secondary cases were defined as those with documented household contact with a confirmed case of the same MLVA type within a time frame that fitted with the incubation period for *Salmonella*. Environmental isolates cultured from food samples or surface swabs during the public health investigation of the two outbreak venues were also included in the analysis. Background information on the two outbreaks was provided by the Communicable Disease Branch and the NSW Food Authority. Isolates were labelled according to a study number and partial laboratory accession number.

### DNA sample preparation

A single colony of each STM isolate was sub-cultured on blood agar plates at 37 °C for 24 h. DNA extraction for MLVA-5 was prepared using a boiling method [23]. For whole genome sequencing, the DNA was extracted and purified using a DNeasy Blood and Tissue Kit (Qiagen) according to the manufacturer’s instructions. DNA quantities were estimated using the Qubit dsDNA HS Assay Kit and the Qubit Fluorometer (Life Technologies, Germany) according to the manufacturer’s instructions.

### MLVA-5

All isolates were MLVA typed using 5 VNTR loci (referred to as MLVA-5) based on an original method by Lindstedt et al. [24] with modifications as described in Wang et al. [23], compatible to the European CDC Scheme [22].

### Genome sequencing

A 100 bp paired-end library (200 cycles) was prepared for each purified DNA sample using the NexteraXT kit (Illumina). Each of the DNA samples was barcoded using the Nextera XT index kit v2 Set A (Illumina) then pooled and sequenced on the HiSeq platform (Illumina, Inc, San Diego, CA, USA) using a single lane of a HiSeq flow cell. Quality control of the libraries was performed by assessing library size distribution on an Agilent 2200 Tapestation (fragment sizes typically range between 250–1000 bp) and libraries were quantified by real time PCR using the KAPA library quantification kit according to the manufacturer’s protocol (Kapa Biosystems). Raw short read sequence data was submitted to Sequence Read Archive (SRA) at the National Centre for Biotechnology Information (NCBI) under the SRA accession number SRP074336.

### SNP analysis

FastQ files were imported into CLC Genomics Workbench v 7.0 (CLC bio, Aarhus, Denmark) and reads were trimmed to remove Nextera transposase adapter sequences then mapped to the reference genome of *S*. Typhimurium LT2 (NCBI GenBank Accession No. NC_003197). Quality-based variant detection was performed using settings of a minimum neighborhood quality of 15 and minimum central quality of 20. Variant detection thresholds were set for a minimum coverage of 10 reads and minimum variant frequency of 75 %. *De novo* assembly was performed on reads from one of the study isolates (isolate 65_E2330) using CLC Genomics Workbench and the resulting contigs were scaffolded using the CLC Microbial Genome Finishing Module. The consensus sequence was extracted from the scaffolded contigs and was then used as a second reference genome for read mapping and variant detection, as described above, to validate SNPs. All predicted SNPs were visually examined by locating the SNP positions in the read-mapping files and comparing the consensus calls to the reference genome. Gene and amino acid allele changes associated with each SNP were determined from the annotated reference genome. We also compared the SNP calls with the method used by us previously [25] and found that the two methods gave highly concordant results with only 2 SNPs not detected in our assembly based method. However, CLC Workbench offers potentially better reproducibility.

### Ethics approval

This study was approved by the Western Sydney Local Health District Human Research Ethics Committee (LNR/14/WMEAD/235, granted 21 July 2014).

## Results

### Isolate selection and outbreak investigation

Of 85 isolates of MLVA 2-15-9-10-0212 recovered from human cases diagnosed between January and May 2014 (inclusive), 56 isolates were epidemiologically classified from the available data and were sequenced in this study. One case was classified as secondary to an outbreak case. A further 10 environmental isolates from the two outbreaks were also included for sequencing. One environmental isolate (65_E2330) from outbreak M had a slightly different but related MLVA profile 2-15-9-9-0212. An isolate with one repeat difference can be regarded as part of this cluster due to rapid evolution at this locus [26].

Following whole genome sequencing, two case isolates from Outbreak A and two from Outbreak M were excluded from the analysis due to the poor quality of sequences. The total number of case isolates included in the study was 52.

**Fig. 1:**
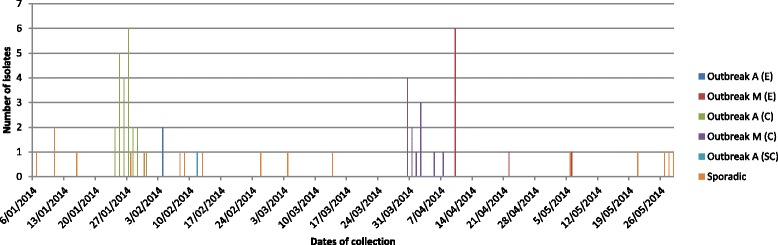
Distribution of isolates by date of collection. Outbreak isolates are labelled as ‘C’ – case, ‘E’ – environmental, or ‘SC’ – secondary case

Outbreak A was linked to chicken liver pate produced weekly from fresh ingredients at a Sydney café. The contaminated ingredient was likely to have been brought in on or around the 25^th^ ﻿of January 2014 as a single event [Food Authority NSW, personal communication, 14 August 2014]. Outbreak M appeared to be caused by contamination of a number of foods and environmental surfaces at a Sydney hot bread shop. All sporadic cases occurred within two months of one or both outbreaks (Fig. 1).

### Whole genome sequencing

#### SNP identification

**Fig. 2:**
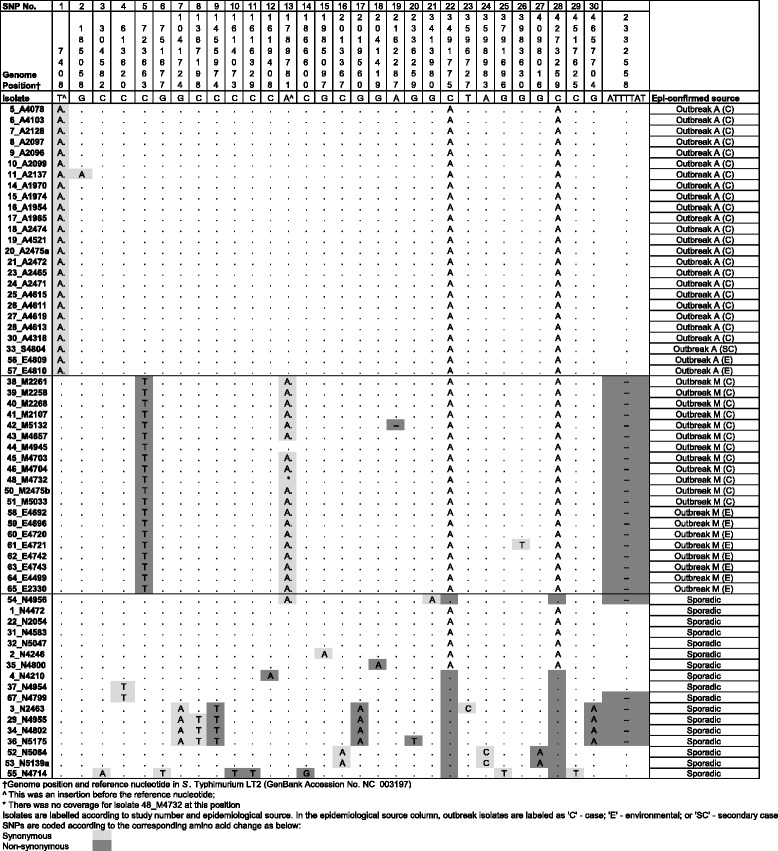
Identification and characterisation of SNPs in the STM isolates. Nucleotides in the main table represent the change from the reference isolate listed with the genome position. Two nucleotide variations represent insertions and are annotated with ^ in the reference position

The genomes were sequenced in one multiplex with an average depth of coverage of 80X ranging from 37X to 154X. A total of 30 SNPs were identified from the 62 isolates. The SNPs called were identical independently of whether the reads were mapped to STM LT2 or to the *de novo* assembly of isolate 65. Based on SNP profiles, all isolates were clustered into groups consistent with their known epidemiological sources (Fig. 2). All case isolates from outbreak M had a unique non-synonymous SNP (C to T) at position 723663 (reference STM LT2 base position), which was also present in the environmental and food isolates obtained from outbreak M. All of the isolates from outbreak M also had a single nucleotide insertion (A) at position 1789781, apart from one case isolate (isolate 44_M4945). In addition, two isolates from outbreak M (one case isolate 42_M5132 and one environmental isolate 61_E4721) carried unique genomic variations at positions 2162287 (a deletion) and 3983630 (a synonymous SNP), respectively.

All of the case and environmental isolates from outbreak A shared a unique single nucleotide insertion (A) at position 7408. One of the case isolates (isolate 11_A2137) also had a unique SNP at position 185058.

The effect of genomic changes on translation was variable. The genomic features of SNPs identified, including the SNP position and type, allele length, annotation, coding region and amino acid changes are provided in the supplementary Additional file 1: Table S1.

Along with SNP differences described above, a seven base-pair deletion at position 2332558 was identified (Fig. 2). This deletion was present in all of isolates from Outbreak M but not those from Outbreak A. The deletion is in *oafA* gene which affects the function of the somatic O antigen of STM – those with the deletion were O antigen factor v (1, 4, 5, i, 1, 2) and those without were O antigen factor xii (1, 4, 12, i, 1, 2) [27].

Sporadic case isolates showed distinct SNP profiles compared to the isolates from Outbreak A and M (Fig. 2). Around half had two additional SNPs (A to C at position 3491775 and 4273259) and there were several unique SNPs randomly distributed among the sporadic isolates. Four cases were distinguished by only one SNP from Outbreak A (isolates 1_N4472, 22_N2054, 31_N4583 and 32_N5047). On examining the epidemiological data, these cases were not linked to Outbreak A. Case 1_N4472 had an onset date well before the introduction of a contaminated ingredient to the Outbreak A venue and 22_N2054 confirmed no link with the venue on interview. Neither 31_N4583 nor 32_N5047 stated any link to Outbreak A during online survey. One of the sporadic isolates (54_N4956) had the same insertion at position 1789781 as the Outbreak M isolates and also had the seven base-pair deletion. However, this isolate also had a SNP at position 3413980 and did not have the other SNPs characteristic of Outbreak M (723663).

Among the sporadic isolates, there were mini-clusters detected by WGS analysis (Fig. 2). One of these clusters contained four cases (3_N2463, 29_N4955, 24_N4802 and 36_N5175), all of which had a common SNP and shared the seven base-pair deletion. The cases occurred over a period of 52 days between January and March 2014. The later three cases shared an additional common SNP and all resided a one geographical area in metropolitan Sydney. Cases in another cluster (52_N5064 and 53_N5139a) shared three common SNPs and were clustered in time (occurring six days apart). In the last cluster (37_N4954 and 67_N4799), the isolates were indistinguishable by SNP analysis, but appeared genetically variable because of the presence of the seven base-pair deletion (different strains with different O antigens). These cases were also separated in time by seven weeks and did not reside in the same geographical area.

**Fig. 3:**
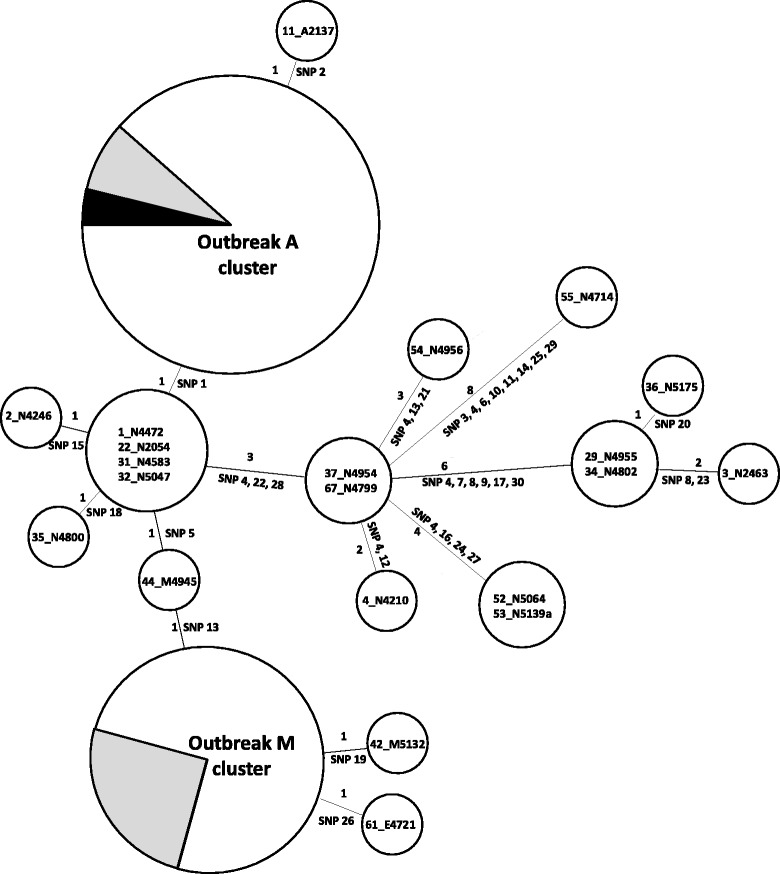
Minimum spanning tree of SNPs identified from the 62 STM isolates. Each circle represents isolates with indistinguishable genome (by SNPs excluding indels) and the size of circles corresponds to the number of isolates. Numbers above or on the left of the connecting lines between circles are the number of SNPs while the SNPs as numbered in Fig. 2 were listed either below or on the right of the connecting lines. Labelling of isolates is consistent with Fig. 2. The grey colour in the outbreak circles indicates the proportion of environmental isolates and the black colour indicates the isolate from the secondary case. Outbreak A cluster included 24 out of 25 isolates (except 11) and Outbreak M cluster included 17 of 20 of the outbreak isolates (except 42, 44, and 61) shown in Fig. 2

Concatenated SNP profiles were further analysed using a minimum spanning tree (MST) approach (Fig. 3). The MST demonstrated that the two outbreaks were clustered separately and that most sporadic cases were more than two SNPs away from the outbreaks and from each other, with only a few exceptions. The four isolates cluster separated by one SNP from outbreak A is actually two nucleotide polymorphism variations when the 7-base pair deletion is included (Fig. 2).

The phylogeny of the outbreak strains was considered in the context of national and international STM isolates. We included 137 isolates from other studies including 75 Australian isolates and 62 global isolates for comparison [15, 21, 25, 28–30]. The phylogenetic tree (Additional file 2: Figure S1) showed that the isolates from this study were tightly clustered together as one group.

## Discussion

Our study examined a collection of STM isolates representing two epidemiologically distinct outbreaks in an urban area along with spatiotemporally associated sporadic cases that were initially linked by MLVA typing. WGS clearly differentiated these isolates in concordance with epidemiological evidence, providing greater discrimination than MLVA-5 alone.

Our study demonstrated unique genomic variations in each of the outbreak clusters, with one or two specific nucleotide variations in each outbreak group. This is consistent with a recent report examining 57 isolates of STM across five outbreaks, where within-outbreak isolates were genetically indistinguishable or differed by one or two SNPs [25]. The findings also independently confirmed those of another recent study that analysed genomes of 12 STM isolates and similarly demonstrated the presence of unique SNPs within outbreaks [21]. In the latter study, however, isolates from distinct outbreaks differed by more than 10 SNPs [21], unlike our two outbreaks, which were more genetically similar. While the isolates in the former study [25] were of the same phage type, they represented five outbreaks in different geographical locations over a period of 3 years, with distinct MLVA types.

Our isolates were all of identical MLVA type and recovered from residents within a confined geographic area of metropolitan Sydney, demonstrating the superior discrimination offered by WGS over current MLVA-5 typing in highly clonal isolates. However, our previous study showed that isolates from the same outbreak may differ by up to 4 SNPs [25]. The low number of genetic differences between outbreak isolates in this study suggests that the two outbreaks may share a common source of origin, such as a single egg manufacturer. The number of SNP difference between isolates observed depends on mutation rate and the evolutionary time that has passed. The mutation rate estimates for *S.* Typhimurium are varied. The lowest rate is 1.9 × 10^7^ substitutions per site per year estimated from ST313 causing invasive infections in Africa, the intermediate rate is 3.4 × 10^7^ substitutions per site per year from epidemic DT104 infections, and the highest rate is 12 × 10^7^ substitutions per site per year from a DT135a outbreak [21, 31, 32]. These rates correspond to an accumulation of approximately one to five SNPs per genome per year. Therefore, the small number of SNP differences observed between our isolates suggests that it is highly likely that these isolates shared a very recent common ancestor.

Other studies have found highly similar isolates within individual outbreaks, along with greater variability between outbreaks, where eggs from the same source were implicated [21, 33]. In NSW, investigation of eggs farms is conducted by the NSW Food Authority and includes MLVA typing of identified *Salmonella* strains. In future investigations, WGS is likely to offer further insights that could make public health investigations more targeted and may allow trace back investigations for a possible common source for outbreaks with closely related isolates such as the outbreaks examined in this study.

Our case outbreak isolates were also largely indistinguishable from the environmental isolates from the same outbreak. Many of our sporadic cases were also well differentiated from the outbreak cases by WGS, and did not carry the unique outbreak variations. However, some sporadic cases showed only minimal differences from the outbreak cases, including the four cases that differed by only one SNP from outbreak A. In addition, one sporadic isolate had the outbreak M insertion and several sporadic isolates contained the 7-bp deletion.

The difference between the outbreak and sporadic isolates was less pronounced than in the previous studies, which demonstrated difference of between 75–100 SNPs [21] to up to several thousand [34] SNPs between sporadic and outbreak isolates. While previous studies included isolates with greater underlying variability, including STM of differing MLVA types collected over a longer time period [21, 34], the small SNP differences between some of our sporadic and outbreak isolates again suggest the possibility of a common environmental (i.e. farm) source. While our sporadic cases clustered separately from outbreak isolates by WGS, the small differences highlight the importance of interpreting epidemiological data alongside WGS findings. The WGS findings suggest that further public health investigation and trace back may be necessary to identify the potential source of both outbreak and sporadic cases.

Unexpectedly, within our sporadic cases, WGS grouped isolates into several apparent and previously undetected mini-clusters. For one of these clusters, epidemiological data indicated the cases were geographically linked. For the others, while the sequencing of these genomes suggested that they were related to each other, public health investigation have not been able to identify any common links between the cases. These findings highlight the need for further comparisons of clusters defined by genomic and public health surveillance and the role of sporadic cases in the natural evolution of outbreaks.

Our report adds to the emerging evidence that WGS challenges traditional epidemiological and typing approaches in the investigation of foodborne outbreaks. For example, previous studies of *Salmonella* Enteritidis and STM suggested the ability of WGS to detect previously unidentified clusters [35] and in a recent study of *Shigella sonnei,* WGS differentiated apparent outbreak strains into three genomically distinct clusters and revealed misclassification by traditional methods [17]. In another recent study in NSW, WGS was able identify STM isolates that were not originally considered to be part of the outbreak [25].

However, genomic surveillance provides only one line of evidence [15] which has be to synthesised with epidemiologic approaches to identify suspected sources [21]. This method, described as ‘the ultimate stain typing system’ [13], has the potential to improve the definition of community outbreaks and assist in a more rapid and targeted public health response through accurate microbiological discrimination of outbreak cases. Recently, a study in the US suggested that WGS of *Salmonella* isolates was feasible for prospective surveillance [35]. In outbreaks of gastroenteritis caused by enterohaemorrhagic *Escherichia coli* (EHEC) in Europe, WGS enabled tracking of outbreak isolates in real-time through the identification of four informative SNPs unique for outbreak isolates [18] and through web-based bioinformatics analysis [14]. The United States Food and Drug Administration, in collaboration with the National Centre for Biotechnology Information and public health laboratories are trialling a centralised surveillance system for WGS data [36]. However, significant challenges in bioinformatics infrastructure, data governance and training of epidemiologists have to be addressed to fully realise benefits of genomics-guided improvements in the delivery of public health surveillance and response [37–39]. A robust and standardised approach to identify potential outbreaks needs to be developed based on the range of SNP differences and the likelihood of those resulting from a recent common ancestor.

A limitation of our study was that we relied on retrospectively well-defined outbreaks. Our epidemiological data, while robust, did not provide sufficient detail for some sporadic cases, which emerged as potentially clustered only following WGS.

## Conclusions

Whole genome sequencing of STM associated with acute gastroenteritis can illuminate distinct foodborne community outbreaks amongst groups of isolates sharing the same MLVA profile. Genomic analyses can also further differentiate sporadic from outbreak cases and cluster sporadic isolates within endemic MLVA types of STM, which can significantly improve the resolution of public health laboratory surveillance. These findings strengthen the case for the adoption of WGS-guided public health surveillance of human salmonellosis and outlines the challenges of distinguishing sporadic cases with minimal differences from outbreak cases.

## Additional files

Additional file 1: Table S1. Genomic features of SNPs identified. (XLS 33 kb) Additional file 2: Figure S1. Phylogeny of the outbreak A and M strains in the context of national and international STM isolates. Genome data analysed in Octavia et al. representing five STM outbreaks in Australia [25]; Kingsley et al. representing ST313 outbreak in Malawi [30]; Leekitcharoenphon et al. representing six STM outbreaks in Denmark [15] and Hawkey et al. representing STM DT135a outbreak in Australia [21] were also included as comparisons and marked as the corresponding study/outbreak. Other branches that are not labelled are background isolates from the above studies; draft genomes from Pang et al. [29] which include five diverse Australian STM isolates; Fu et al. representing *Salmonella* reference collection A; [28] and other fully sequenced STM genomes available from GenBank including LT2 (Accession No. NC003197), 798 (Accession No. CP003386), DT2 (Accession No. HG326213), DT104 (Accession No. HF937208), 14028S (Accession No. CP001363), SL1344 (Accession No. FQ312003), UK-1 (Accession No. CP002614), T000240 (Accession No. AP011957), U288 (Accession No. CP003836) and ST4/74 (Accession No. CP002487). Bootstrap values if greater than 50 %, are presented on the internal branches. (PPTX 74 kb)

## Abbreviations

ICPMR: Institute for Clinical Pathology and Medical Research
NCIMS: Notifiable Conditions Information Management Systems
SNP: Single nucleotide polymorphism
STM: *Salmonella* Typhimurium
WGS: Whole genome sequencing.
