# Supplementary figures and images for: Whole genome sequencing of Salmonella Typhimurium illuminates distinct outbreaks caused by an endemic multi-locus variable number tandem repeat analysis type in Australia, 2014
Source: BMC Microbiol. 2016 Sep 15;16:211. doi: 10.1186/s12866-016-0831-3 (PMC5024487; doi:10.1186/s12866-016-0831-3)

## Slide 1
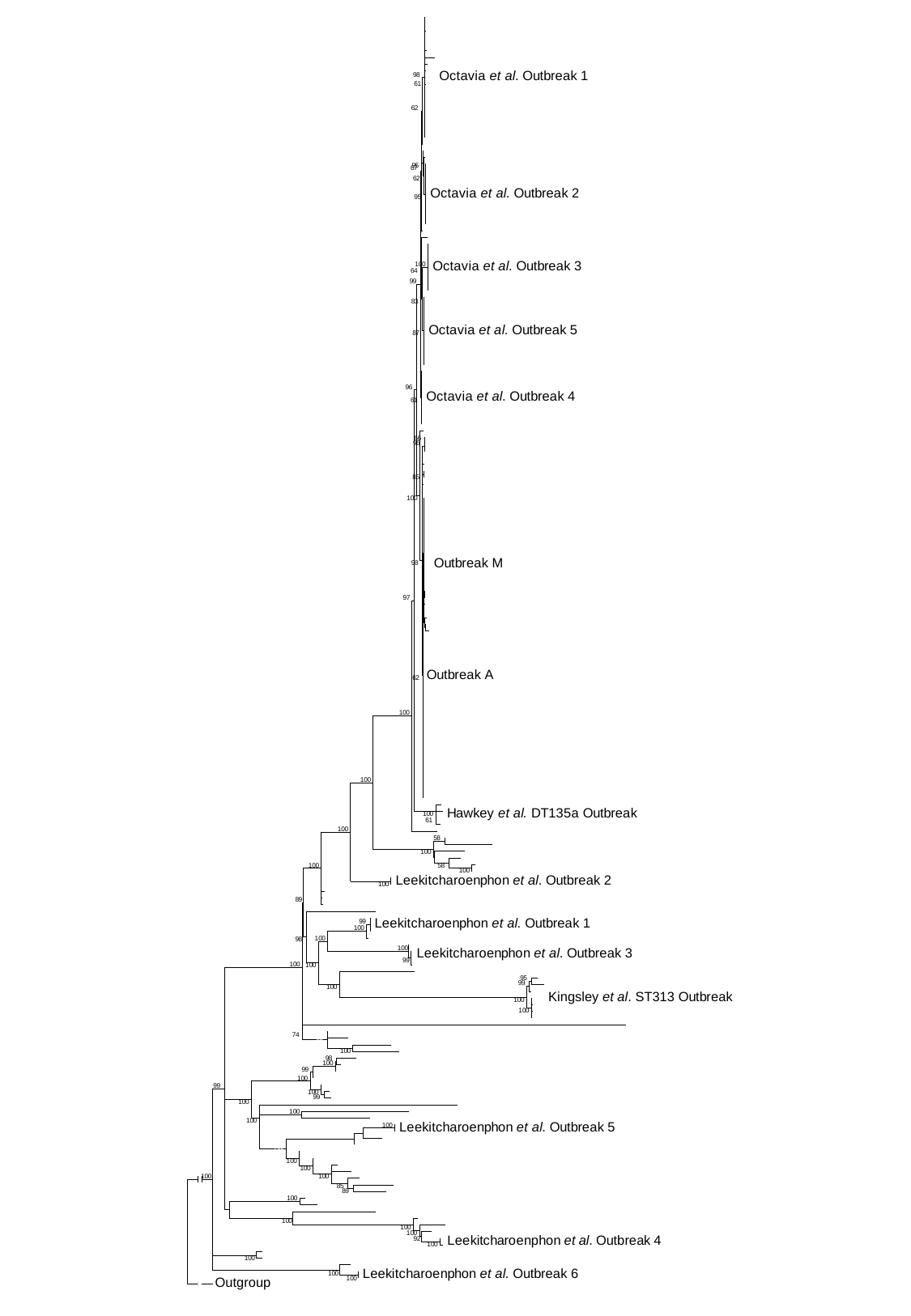

Supplement: Additional file 2: Figure S1. — Phylogeny of the outbreak A and M strains in the context of national and international STM isolates. Genome data analysed in Octavia et al. representing five STM outbreaks in Australia [25]; Kingsley et al. representing ST313 outbreak in Malawi [30]; Leekitcharoenphon et al. representing six STM outbreaks in Denmark [15] and Hawkey et al. representing STM DT135a outbreak in Australia [21] were also included as comparisons and marked as the corresponding study/outbreak. Other branches that are not labelled are background isolates from the above studies; draft genomes from Pang et al. [29] which include five diverse Australian STM isolates; Fu et al. representing Salmonella reference collection A; [28] and other fully sequenced STM genomes available from GenBank including LT2 (Accession No. NC003197), 798 (Accession No. CP003386), DT2 (Accession No. HG326213), DT104 (Accession No. HF937208), 14028S (Accession No. CP001363), SL1344 (Accession No. FQ312003), UK-1 (Accession No. CP002614), T000240 (Accession No. AP011957), U288 (Accession No. CP003836) and ST4/74 (Accession No. CP002487). Bootstrap values if greater than 50 %, are presented on the internal branches. (PPTX 74 kb) [file 12866_2016_831_MOESM2_ESM.pptx]
